# Computational Modeling of Interstitial Fluid Pressure and Velocity in Non-small Cell Lung Cancer Brain Metastases Treated With Stereotactic Radiosurgery

**DOI:** 10.3389/fneur.2020.00402

**Published:** 2020-05-28

**Authors:** Nathaniel Swinburne, Eve LoCastro, Ramesh Paudyal, Jung Hun Oh, Neil K. Taunk, Akash Shah, Kathryn Beal, Behroze Vachha, Robert J. Young, Andrei I. Holodny, Amita Shukla-Dave, Vaios Hatzoglou

**Affiliations:** ^1^Department of Radiology, Memorial Sloan Kettering Cancer Center, New York, NY, United States; ^2^Department of Medical Physics, Memorial Sloan Kettering Cancer Center, New York, NY, United States; ^3^Department of Radiation Oncology, Hospital of the University of Pennsylvania, Philadelphia, PA, United States; ^4^Department of Radiation Oncology, Memorial Sloan Kettering Cancer Center, New York, NY, United States

**Keywords:** Stereotactic radiosurgery (SRS), brain metastases from lung cancer, perfusion MRI, computational fluid modeling, interstitial fluid pressure, treatment response

## Abstract

**Background:** Early imaging-based treatment response assessment of brain metastases following stereotactic radiosurgery (SRS) remains challenging. The aim of this study is to determine whether early (within 12 weeks) intratumoral changes in interstitial fluid pressure (IFP) and velocity (IFV) estimated from computational fluid modeling (CFM) using dynamic contrast-enhanced (DCE) MRI can predict long-term outcomes of lung cancer brain metastases (LCBMs) treated with SRS.

**Methods:** Pre- and post-treatment T_1_-weighted DCE-MRI data were obtained in 41 patients treated with SRS for intact LCBMs. The imaging response was assessed using RANO-BM criteria. For each lesion, extravasation of contrast agent measured from Extended Tofts pharmacokinetic Model (volume transfer constant, K^trans^) was incorporated into a computational fluid model to estimate tumor IFP and IFV. Estimates of mean IFP and IFV and heterogeneity (skewness and kurtosis) were calculated for each lesion from pre- and post-SRS imaging. The Wilcoxon rank-sum test was utilized to assess for significant differences in IFP, IFV, and IFP/IFV change (Δ) between response groups.

**Results:** Fifty-three lesions from 41 patients were included. Median follow-up time after SRS was 11 months. The objective response (OR) rate (partial or complete response) was 79%, with 21% demonstrating stable disease (SD) or progressive disease (PD). There were significant response group differences for multiple posttreatment and Δ CFM parameters: post-SRS IFP skewness (mean −0.405 vs. −0.691, *p* = 0.022), IFP kurtosis (mean 2.88 vs. 3.51, *p* = 0.024), and IFV mean (5.75e-09 vs. 4.19e-09 m/s, *p* = 0.027); and Δ IFP kurtosis (mean −2.26 vs. −0.0156, *p* = 0.017) and IFV mean (1.91e-09 vs. 2.38e-10 m/s, *p* = 0.013). Posttreatment and Δ thresholds predicted non-OR with high sensitivity (sens): post-SRS IFP skewness (−0.432, sens 84%), kurtosis (2.89, sens 84%), and IFV mean (4.93e-09 m/s, sens 79%); and Δ IFP kurtosis (−0.469, sens 74%) and IFV mean (9.90e-10 m/s, sens 74%).

**Conclusions:** Objective response was associated with lower post-treatment tumor heterogeneity, as represented by reductions in IFP skewness and kurtosis. These results suggest that early post-treatment assessment of IFP and IFV can be used to predict long-term response of lung cancer brain metastases to SRS, allowing a timelier treatment modification.

## Introduction

Brain metastases (BMs) represent the largest category of intracranial malignant tumors with an annual incidence 3–10 times greater than primary brain malignancies (1, 2). Occurring in up to 40% of patients with systemic cancer (3), BMs represent a major source of morbidity and mortality in this population. In particular, the most frequent source are primary lung malignancies, which comprise up to 36–64% of brain metastases (4).

Radiation therapy (RT) is the standard of care for patients in whom complete surgical resection is not possible due to surgically inaccessible lesion locations, disqualifying comorbidities, or uncontrolled systemic disease. Stereotactic radiosurgery (SRS), which employs single high-dose targeted treatment using stereotactic image guidance, has shown comparable efficacy to whole brain radiation therapy (WBRT) in controlling oligometastatic intracranial disease, achieving >80–90% local control while decreasing the risks of toxicities, such as neurocognitive decline (5–8).

Identification of eventual non-response in the early post-SRS time period is difficult. Surveillance MRI, which represents the standard for assessing brain metastasis treatment response, may be confounded in the early post-treatment setting by the tendency of up to one-third of BMs to transiently increase in size following SRS (9). The ability to predict SRS failure has major clinical importance, as it would potentially allow non-responsive tumors to undergo treatment intensification or prompt modifications to systemic therapy regimens.

The limitations of traditional size-based treatment response assessment following locoregional therapies, such as SRS, have driven the development of advanced MR imaging techniques, such as dynamic contrast-enhanced (DCE) perfusion MRI, that go beyond anatomic visualization to characterize tumor physiology. The extended Tofts pharmacokinetic model (ETM) is one such paradigm that quantifies surrogate measures of vascular permeability (i.e., volume transfer constant, K^trans^ [min^−1^]). Interstitial pressures within the tumor affect the extravasation of medications into the interstitium and influence the response and outcome to radiotherapy (10–12).

The disorganized and tortuous architecture of blood vessels results in altered fluid dynamics across the vasculature and in the interstitium. The resulting elevated interstitial fluid pressure (IFP) effectively reduces the hydrostatic pressure differential that normally exists between vasculature and extracellular extravascular spaces, which can adversely impact the successful delivery of anti-tumor therapy (13–15). IFP returns to normal levels in the healthy tissues surrounding the tumor. The precipitous drop in IFP at the tumor periphery results in a zone where the interstitial fluid velocity (IFV) is increased and directed outward, causing rapid exudate flux of interstitial fluid from regions of high to low pressure (16), further diminishing the effectiveness of drug delivery and therapy.

To support this model, the direct invasive measurement of intralesional IFP in cervical cancer using modified wick-in-needle (WIN) probes has shown mid–radiation therapy IFP to be significantly different between patients with complete and partial responses at 1 month post-treatment (17). Fyles et al. reported high IFP measurement to be associated with a negative prognosis in cervical cancer (18). However, the invasive measurement of IFP and interstitial fluid velocity (IFV), which can be derived from the IFP gradient (19), is not feasible in many settings, especially where a tumor cannot be easily or safely accessed. Additionally, single-point WIN probing of tumor does not provide insight into the internal spatial variation of IFP. Therefore, non-invasive computational fluid modeling (CFM) to provide estimates of tumor IFP using the volume transfer constant (K^trans^) obtained from ETM (20, 21) is a desirable alternative. K^trans^ is incorporated into an observable CFM equation to modulate the net pressure developed in tissue, including trans-capillary hydrostatic pressure, for the delivery of fluid, which is taken into consideration by conventional DCE-derived pharmacokinetic models.

Previously, we investigated the ability of ETM parameters to predict long-term local tumor control in the early post-SRS setting for patients with lung cancer brain metastases. We showed that K^trans^ standard deviation (SD) was highly sensitive (89%) for predicting disease progression vs. no progressive disease (22). This result was not surprising, as K^trans^ SD is considered a marker of tumor vascular heterogeneity (22, 23), and tumors are known to recruit disorganized and heterogeneous microvasculature.

In the present study, we have aimed to investigate whether non-invasive IFP and IFV estimates of global tissue physiology can predict the long-term response of lung cancer brain metastases treated within 12 weeks of SRS. The patient cohort is from our previous work that utilized more conventional DCE-derived parameters. These novel imaging biomarkers may further our ability to optimize patient management by demonstrating changes in tumor physiology.

## Materials and Methods

### Patients and Treatment

This retrospective investigation was performed at a tertiary cancer center following institutional review board approval and in accordance with the Health Insurance Portability and Accountability Act. Patients treated between 2012 and 2015 that met the following inclusion criteria were included in this analysis:

- Histopathologic diagnosis of non-small cell lung cancer (NSCLC).
- Treatment of one or more intact (non-resected) brain metastases with SRS.
- No history of WBRT prior to SRS.
- DCE perfusion MRI scans, including coverage of the treated lesion(s) obtained both pre-treatment and within 12 weeks following SRS treatment.

Patient demographic and treatment data collected included patient age, histologic tumor subtype, three-dimensional lesion size, lesion location, and SRS treatment dose(s). All treatments employed single-fraction SRS. Our study population consisted of 41 patients who were previously included in a study analyzing DCE-MRI parameters (22).

### MR Perfusion Imaging Acquisition

Patients were scanned on 1.5T or 3T scanners (Signa Excite, HDx and Discovery 750, GE Healthcare) using an 8-channel head coil. Standard T1-weighted, T2-weighted, diffusion-weighted, fluid-attenuated inversion recovery, susceptibility-weighted, and contrast T1-weighted images were acquired in multiple planes. T1-weighted DCE data were acquired using an axial 3D spoiled gradient-echo sequence (repetition time [TR], 4–5 ms; echo time [TE], 1–2 ms; section thickness, 5 mm; flip angle [FA], 25 degrees; field of view, 24 cm; matrix, 256 × 128). Ten phases were acquired pre-injection followed by another 30 phases during the dynamic injection of intravenous contrast. This was followed by a 40-mL saline flush. The time between phases (temporal resolution) was 5–6 s. Matching contrast T1-weighted (TR/TE, 600/8 ms; thickness, 5 mm; matrix, 256 × 224) spin-echo images were obtained. Ten to twelve slices were obtained to cover the entire volume of each lesion. Gadopentetate dimeglumine (Magnevist; Bayer HealthCare Pharmaceuticals, Berlin, Germany) was power-injected via an intravenous catheter (18–21 gauge) at doses standardized by patient body weight (0.2 mL/kg body weight, maximum 20 mL) at 2–3 mL/s. High resolution 3D T1-weighted contrast-enhanced images in the axial plane with a slice thickness of 1 mm and no gaps between slices were routinely acquired for SRS planning and follow-up after therapy.

### DCE MRI Pharmacokinetic Modeling

The two-compartment extended Tofts model (ETM) accounts for vascular space (*v*_p_) and extravascular extracellular space [EES], (*v*_e_). The ETM expression for modeling *C*_t_(*t*) is given (24):

(1) $$\begin{array}{l} C_{\text{t}}\left(t\right)=K^{\text{trans}}\overset{\text{t}}{\underset{0}{\int }}{e^{-k_{\text{ep}}\left(t-\tau \right)}C}_{\text{p}}\left(\tau \right)d\tau +\;{v_{\text{p}}C}_{\text{p}}\left(t\right) \end{array}$$

where, *K*^trans^ (min^−1^) is the volume transfer constant of CA, *C*_p_(*t*) is the delivery time-course of plasma CA concentration (or arterial input function, AIF), and *v*_e_ and *v*_p_ are the volume fractions of the EES and blood plasma, respectively. CA transfer from EES back into the vascular space is defined as *k*_ep_ = *K*^trans^/*v*_e_. A detailed calculation of tissue contrast concentration *C*_t_ from DCE signal is given elsewhere (25).

AIF for each patient was selected from a sagittal sinus voxel in the imaging time course (25, 26). Brain metastasis volumes-of-interest (VOIs) were manually segmented by an attending neuroradiologist on late phases of the T_1_w DCE images using ITK-SNAP (27).

In the absence of multi-flip angle pre-contrast T_1_ images, T_10_ values were set to either 0.8 or 1s (dependent on magnetic field strength) for each voxel in determination of Δ*R*_1_. Tissue concentration time course, *C*_t_(*t*) [Equation. (1)], was calculated using non-linear fitting to minimize the sum of squared errors (SSE) between model fit and data. The fitting procedure estimates the values of *K*^trans^, *v*_e_ and *v*_p_, for each voxel. Parameter estimation bound limits were set: *K*^trans^ ε [0, 5] (min^−1^), *v*_e_, and *v*_p_ ε [0, 1]. All DCE data analysis was performed using in-house MRI-QAMPER software (Quantitative Analysis Multi-Parametric Evaluation Routines) written in MATLAB (The MathWorks, Inc., Natick, MA).

### Interstitial Fluid Pressure Simulation

#### CFM Mathematical Model

The fluid mechanics of a system are given by the Navier-Stokes hydrodynamic mass-balance equation (28). The extracellular matrix is modeled as a porous medium. We assumed the case of an incompressible fluid, ignoring friction within fluid, and exchange of momentum between fluid and solid phases. Fluid movement through EES is approximated with low-Reynolds Number flow (29) and modeled under assumption of steady-state velocity. We applied the well-known Darcy's Law to describe bulk fluid movement, expressing the product of gradient in IFP (∇*p*_*i*_) and the hydraulic conductivity of the porous medium, *K*_H_ as the interstitial fluid velocity, **u**:

(2) $$\begin{array}{l} \text{u}=-K_{\text{H}}\nabla p_{i} \end{array}$$

A dynamic system can then be modeled as follows: fluid enters EES via the vascular compartment. In the human brain, there is no established lymphatic system of clearance, and we take the lymphatic drainage function to be zero in both normal and tumor tissue.

The full derivation for the continuity equation [Equation. 3] is presented in the Appendix. The final form is given in terms of the dependent variable interstitial pressure, *p*_*i*_:

(3) $$\begin{array}{l} -K_{\text{H}}\nabla^{2}p_{\text{i}}=\frac{K^{\text{trans}}}{\langle K^{\text{trans}}\rangle }[L_{\text{P}}\frac{S}{V}\left(p_{\text{V}}-p_{\text{i}}-\sigma_{\text{T}}\left(\pi_{\text{V}}-\pi_{\text{i}}\right)\right)]\\ -\frac{L_{\text{pL}}S_{\text{L}}}{V}\left(p_{\text{i}}-p_{\text{L}}\right) \end{array}$$

where 〈*K*^trans^〉 represents mean *K*^trans^ values within the tumor; this term is used to account for heterogeneous fluid leakiness in the tumor (30), *L*_*P*_ is the hydraulic conductivity of the capillary wall (or vessel permeability), *S/V* is microvascular surface area per unit volume, *p*_V_ is the blood pressure in the microvessel, and *p*_i_ is interstitial fluid pressure; π_V_ is osmotic pressure in microvasculature, π_i_ is osmotic pressure in interstitial space, and σ_*T*_ is the osmotic reflection coefficient. The lymphatic clearance term, *L*_pL_*S*_L_/*V*, is assumed to be zero in the brain. Estimates for physical parameters were selected in agreement with previous literature on modeling IFP in brain tumors (21, 31–34).

#### Computational Fluid Modeling

The continuity partial differential equation (PDE) was implemented using the COMSOL CFM simulation PDE module. Solving [Equation. 7] provides the basis for estimation of *p*–_i_ and 3D parametric maps of IFP and IFV.

The 3D physiological mesh model was generated from each patient's T_1_w DCE tumor images. ROIs for tumor in the simulation domain were resliced to be 1 mm^3^-isotropic in MATLAB using the NIfTI Toolbox (35) and converted to stereolithography (STL) file format. STL files were imported into the simulation software and interpreted as boundary meshes for the model.

ETM-estimated *K*^trans^ maps were co-registered to match the simulation mesh space. *K*^trans^ maps were incorporated in COMSOL as a scalar field over the simulation domain and numerical values for physical constants in normal and tumor tissue were defined in the appropriate regions of the 3D STL domain mesh, as listed in Table 1. A stationary solution of Equation 13 was computed on the 3D extended domain ROI, and pressure at the simulation boundary was set to zero to agree with pressure conditions in normal brain tissue.

**Table 1:** List of assigned physical parameters in the CFM simulation.

**Parameter**	**Description**	**Units**	**Normal tissue**	**Tumor tissue**	**References**
*K*_H_	Interstitial hydraulic conductivity	$\frac{{\text{m}}^{2}}{\text{Pa }\cdot \text{s}}$	5.65 × 10^−15^	4.9 × 10^−13^	(21, 36, 37)
*L*_p_	Vascular hydraulic conductivity	$\frac{{\text{m}}^{2}}{\text{Pa }\cdot \text{s}}$	8 × 10^−14^	6.4 × 10^−13^	(21, 38, 39)
S/V	Vessel exchange area	m^−1^	10,000	20,000	(21, 31)
*P*_eff_	Effective pressure	Pa	400	1,550	(21, 31)

Simulation was conducted using the general coefficient form PDE module in a commercial multiphysics software package (COMSOL Inc., Stockholm, Sweden) using the finite element method to solve PDE computations.

### Data Analysis

We analyzed IFP and IFV parameters as follows. For each metric, we computed the mean and descriptive statistics such as standard deviation (SD), kurtosis, and skewness, leading to a set of eight features. We generated three sets of these eight features: pre-RT, post-RT, and the change of values (denoted as Δ) between pre-RT and post-RT. For each imaging feature, an average value was computed across multiple slices on each lesion. Univariate analysis was performed using the Wilcoxon rank-sum test to find the degree of differences in these features between patients with an objective response (either partial or complete response, OR) vs. non-OR (either stable disease or progressive disease). A receiver operating characteristic (ROC) curve analysis was performed to find the best cutoffs on these features using Youden's index.

For clinical data, local control was assessed by the modified Response Assessment in Neuro-Oncology Brain Metastases (RANO-BM) criteria using conventional MRI (40), with additional information from surgical resection if performed after SRS. The modification we made to standard RANO-BM criteria was to lower the minimum size limit of measurable disease to 5 mm. We chose to lower the limit because we routinely treat BMs measuring between 5 and 10 mm with SRS at our institution. Local relapse-free survival was calculated from day of treatment to most recent imaging. Failure was determined by progressive disease defined by RANO-BM or surgical resection indicating viable tumor. All statistical analysis was performed using R language version 3.5.2 and MATLAB version R2018b.

## Results

Forty-one patients with 53 BMs were included in the analysis (Table 2). Thirty-two patients had a single brain metastasis, seven patients had two metastases, and two patients had three or more metastases. Median SRS treatment dose was 21 Gy (range, 18–22 Gy).

**Table 2:** Patient characteristics.

**Characteristic**		***N* (%)**
Number	Patients	41
	Lesions	53
Sex	Male	21 (51.2)
	Female	20 (48.8)
Age (y)	Median (range)	52 (36–71)
Histologic subtype (by patient)	Adenocarcinoma	35 (85.4)
	Squamous cell	3 (7.3)
	Large cell	1 (2.4)
	Poorly differentiated/not otherwise specified	2 (4.9)
Number of lesions	Single	29 (70.7)
	Multiple	12 (29.3)
Location	Supratentorial	46 (86.8)
	Infratentorial	7 (13.2)
Radiation dose	Median (range)	21 Gy (18–22)

No patients received concurrent systemic therapy with SRS. Per our institution's general practice, most patients (*n* = 39) had a washout period before and following SRS: at least 2 weeks for systemic therapies and at least 1 week for targeted systemic therapies.

Post-treatment imaging was obtained 7–8 weeks (*n* = 19), 9–10 weeks (*n* = 16), or 11–12 weeks (*n* = 18) following SRS. Median duration of post-treatment follow-up was 11 months (range, 3.7–8.3 months), including 73% of patients who were followed until death. Following SRS, one patient subsequently underwent resection for a growing mass and was found to have viable tumor. Eight patients were treated with WBRT after SRS for control of non-index metastases. Local control at 1-year post-treatment was 85% as determined by subsequent histopathologic sampling, where available, or RANO-BM imaging criteria. Rates of complete response, partial response, stable disease, and progressive disease were 9, 49, 21, and 21%, respectively (Table 3).

**Table 3:** Summary of RANO-BM response categories.

	**RANO-BM category**	**Number of lesions**
OR	CR	5 (9%)
	PR	26 (49%)
Non-OR	SD	11 (21%)
	PD	11 (21%)

*OR, objective response; CR, complete response; PR, partial response; SD, stable disease; PD, progression of disease*.

Univariate analysis using Wilcoxon rank-sum test (Table 4) showed a significant difference between lesions showing OR vs. non-OR: post-SRS IFP skewness (mean −0.405 vs. −0.691, *p* = 0.022), IFP kurtosis (mean 2.88 vs. 3.51, *p* = 0.024), and IFV mean (5.75e-09 vs. 4.19e-09 m/s, *p* = 0.027); and Δ IFP kurtosis (mean −2.26 vs. −0.0156, *p* = 0.017) and IFV mean (1.91e-09 vs. 2.38e-10 m/s, *p* = 0.013). Using the Youden index, balanced thresholds for differentiating non-OR vs. OR were determined: post-SRS IFP skewness −0.432 (sensitivity 84%, specificity 59%), IFP kurtosis 2.89 (sensitivity 84%, specificity 63%), and IFV mean 4.93e-09 m/s (sensitivity 79%, specificity 67%); and Δ IFP kurtosis −0.469 (sensitivity 74%, specificity 59%) and IFV mean 9.90e-10 m/s (sensitivity 74%, specificity 74%). SRS dose was not significantly correlated with RANO-BM treatment response.

**Table 4:** Univariate analysis using the Wilcoxon rank-sum test.

	**Parameter (Units)**	**Non-OR mean**	**OR mean**	***P***
Pre-SRS	IFP mean (kPa)	1.44	1.44	0.784
	IFP SD (kPa)	0.0139	0.0257	0.752
	IFP skewness	−0.698	−6.33	0.430
	IFP kurtosis	3.53	4.93	0.644
	IFV mean (m/s)	3.95e-09	3.80e-09	0.774
	IFV SD (m/s)	2.56e-09	2.44e-09	0.518
	IFV skewness	1.79	1.72	0.926
	IFV kurtosis	9.39	8.95	0.782
Post-SRS	IFP mean (kPa)	1.40	1.42	0.705
	IFP SD (kPa)	0.0145	0.0178	0.186
	IFP skewness	−0.691	−0.405	**0.0216**
	IFP kurtosis	3.51	2.88	**0.0243**
	IFV mean (m/s)	4.19e-09	5.75e-09	**0.0272**
	IFV SD (m/s)	2.61e-09	3.10e-09	0.265
	IFV skewness	1.61	1.28	0.135
	IFV kurtosis	8.24	6.47	0.228
Change	IFP mean (kPa)	−0.0475	−0.0154	0.428
	IFP SD (kPa)	5.91e-04	−9.55e-03	0.441
	IFP skewness	0.00614	0.357	0.255
	IFP kurtosis	−0.0156	−2.26	**0.0170**
	IFV mean (m/s)	2.38e-10	1.91e-09	**0.0132**
	IFV SD (m/s)	5.33e-11	5.80e-10	0.177
	IFV skewness	−0.179	−0.577	0.141
	IFV kurtosis	−1.16	−3.12	0.188

*OR, objective response; IFP, interstitial fluid pressure; IFV, interstitial fluid velocity; SD, standard deviation. Bolded values represent statistically significant P-values (P < 0.05)*.

**Figure 1:**
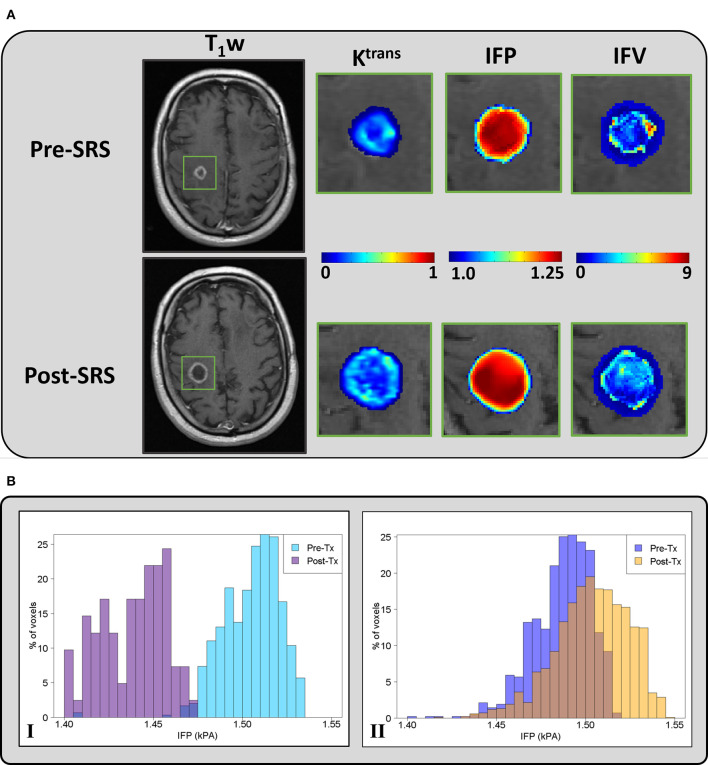
**(A)** - Representative pre-SRS and post-SRS T1-weighted post-contrast MR images of a patient (59 years, male) with brain metastases who experienced progressive disease (PD). The green rectangle delineates the ROI at the metastatic lesion. K^trans^ (min^−1^), IFP (kPa), and IFV (10^−9^ m/s) maps are zoomed at the location of the ROI. **(B)** - Histograms of percentage (%) voxel-wise IFP values at pre- and post-SRS treatment from representative patients who experienced (I) OR (male, 73 years old) and (II) non-OR (male, 47 years old).

**Figure 2:**
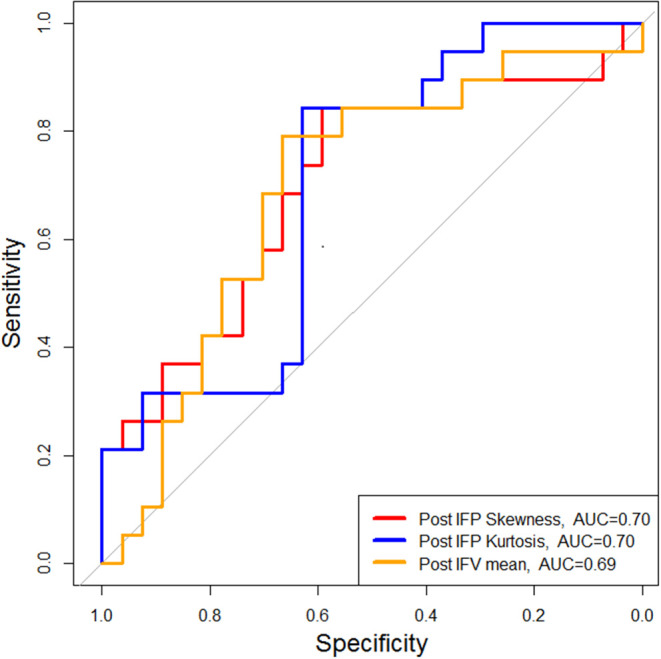
Patients with OR showed significantly lower mean IFP skewness and kurtosis and higher mean IFV within 12 weeks post-SRS compared with patients with non-OR (either SD or PD). OR, objective response; SD, stable disease; PD, progressive disease.

**Figure 3:**
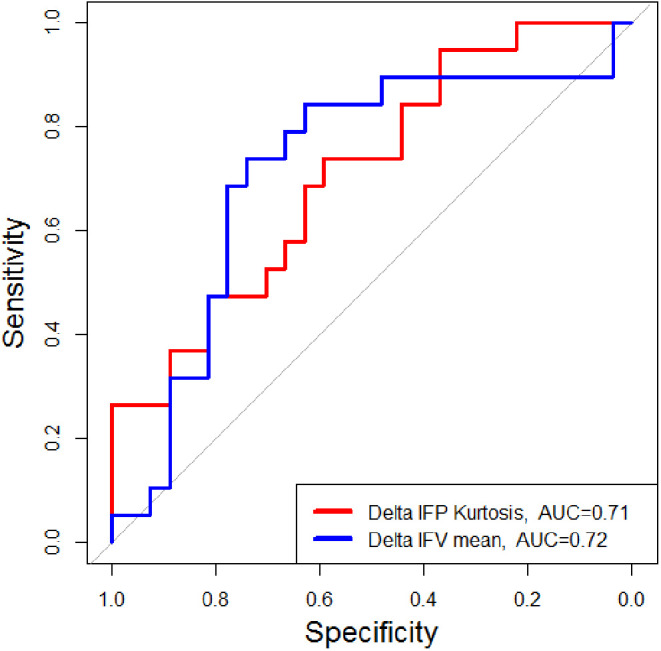
Patients with OR showed significantly greater pre-SRS to post-SRS (Δ) declines in mean IFP kurtosis and greater increases in mean IFV within 12 weeks post-SRS compared with patients with non-OR (either SD or PD). OR, objective response; SD, stable disease; PD, progressive disease.

Figure 1A shows representative pre-SRS and post-SRS anatomic MR images and corresponding lesion K^trans^, IFP, and IFV color maps. Figure 1B exhibits representative histograms showing the distribution of intratumoral voxel values for IFP in two patients who experienced OR vs. non-OR. The left-shift in the IFP values (kPa) of the histogram for the patient who experienced OR shows a decrease in pressure after treatment. In contrast, the histogram of a patient who experienced non-OR shows a subtle upward shift in mean IFP values and has many voxels with the same IFP value, resulting in a skew distribution. Receiver operating characteristic areas-under-curve for post-SRS IFP skewness (0.70), IFP kurtosis (0.70), and IFV mean (0.69); and Δ IFP kurtosis (0.71) and IFV mean (0.72) are shown in Figures 2, 3.

## Discussion

We investigated whether non-invasive estimates of intratumoral IFP and IFV in the early post-treatment setting using ETM-derived K^trans^ can predict the long-term response of lung cancer brain metastases to SRS. IFP and IFV parameters estimated from CFM were able to accurately predict long-term response using both isolated post-treatment values (IFP kurtosis, IFP skewness, and IFV mean) and changes between post- and pre-treatment values (Δ IFP kurtosis and Δ IFV mean). Our results support the use of these biomarkers as early post-SRS predictors of long-term treatment response in LCBMs. These parameters may enable the earlier identification of LCBM non-responders, allowing more timely treatment intensification or modifications to systemic therapies.

A major cause of elevated IFP within tumors is aberrant microvasculature resulting in altered fluid dynamics (41). Radiation therapy causes early and sustained damage to vasculature (42–44), which tends to lower intratumoral microvascular heterogeneity (45). In our cohort, IFP kurtosis and skewness, measures of tumoral IFP heterogeneity, showed early decreases in patients who ultimately showed objective response to SRS. This is intuitive, since post-treatment necrosis would be expected to smooth tumoral IFP distribution, resulting in decreased heterogeneity. Furthermore, Smith et al. showed that a necrotic tumor core, which lacks functioning vasculature necessary for fluid resorption, results in decreased pressure decay within the core and thus promotes increased IFV (31). This may provide a framework to explain why IFV mean was both significantly higher following SRS and showed greater relative increases from baseline in patients with objective response. Conversely, the correlation between poor outcomes and persistent intratumoral hypertension following treatment may be related to previous observations that high IFP in extracranial tumors decreases the uptake of chemotherapy drugs (41, 46) and promotes the outward flow of tumor-promoting growth factors and chemoreceptor ligands (47, 48).

To the best of our knowledge, ours is the first study to assess the predictive capabilities of non-invasively estimated IFP and IFV parameters for brain metastases that have been treated with SRS. A prior investigation successfully used these parameters to predict outcomes in cervical cancer (49). Utilizing histogram analysis, we showed a difference in the distribution of IFP in patients who experienced OR vs. non-OR. Similar to prior studies in cervical cancer (17, 50), IFP may be a useful prognostic indicator for brain metastases. Further investigation is needed to determine whether the evaluation of metastasis microenvironments from CFM estimated IFP and IFV can be utilized to personalize therapy regimens and improve outcomes. This may be especially relevant in the context of targeted chemotherapeutic agents and immunotherapy since elevated intratumoral IFP can prevent adequate penetration of intravenous drugs.

Boucher et al. (51) reported direct WIN measurements of IFP in rodent models, and from 11 human primary brain tumors during intracranial brain surgery. The rodent brain tumor mean IFP in *n* = 4 small F98 gliomas (V_mean_ = 10 ± 2.5 mm^3^) was 1.2 + 0.33 kPa. In 10 of the 11 human cases, IFP ranged from 0.066 to 0.4 kPa; in one astrocytoma, IFP was found to be 1.2 kPa. The estimates of IFP in our CFM are consistent with the measured results in the small-scale rodent tumors. Similar results were found in measurements of IFP in preclinical study by Navalitloha et al. (52) on rat gliomas.

A strength of our study is the inclusion of only NSCLC brain metastases undergoing single modality locoregional therapy with SRS. This allows for the relative control of potential confounders, including heterogeneous tumor histologies and variable baseline treatment effects from non-ablative modalities like WBRT. Additionally, the majority of our patients were followed until death, providing a clearer understanding of individual lesion outcomes.

As a retrospective investigation limited to BMs of a single, albeit common, histopathology, our results cannot be generalized to the treatment of BMs with SRS more broadly. With respect to our fluid model, the parameter values for hydraulic conductivity, vessel permeability, effective pressure, and microvascular surface area need to be verified experimentally to increase the simulation accuracy. Furthermore, the lack of confirmatory direct lung cancer brain metastasis pressure measurements within our cohort, for example, via the WIN approach, precludes the comparison of our derived IFP and IFV estimations against a gold standard.

In conclusion, this study shows that IFP and IFV parameters in lung cancer brain metastases derived from DCE-MRI within 12 weeks of SRS can predict long-term local tumor control. These results suggest that IFP and IFV represent promising imaging biomarkers that can non-invasively characterize global tissue physiology in lung cancer brain metastases. Further investigation is needed to validate these results for other brain metastasis histologies and to assess the use of these non-invasive biomarkers to guide personalized treatment regimens that target the tumor microenvironment.

## Data Availability Statement

The datasets generated for this study will not be made publicly available The source data contain inherent protected health information. Requests to access the data are directable to the corresponding author.

## Ethics Statement

The studies involving human participants were reviewed and approved by Memorial Sloan Kettering Cancer Center Institutional Review Board. Written informed consent for participation was not required for this study in accordance with the national legislation and the institutional requirements.

## Author Contributions

VH, AS-D, and EL conceived the idea. NS, VH, EL, RP, and AS-D collected and analyzed data and wrote the manuscript. All authors discussed results and made substantive changes to the final manuscript.

## Conflict of Interest

The authors declare that the research was conducted in the absence of any commercial or financial relationships that could be construed as a potential conflict of interest. The handling editor is currently organizing a Research Topic with one of the authors BV.
