# Supplementary material for: Computational Modeling of Interstitial Fluid Pressure and Velocity in Non-small Cell Lung Cancer Brain Metastases Treated With Stereotactic Radiosurgery
Source: Front Neurol. 2020 May 28;11:402. doi: 10.3389/fneur.2020.00402 (PMC7271672; doi:10.3389/fneur.2020.00402)
Supplement: Supplementary file 1 [file Data_Sheet_1.pdf]

**Computational modeling of interstitial fluid pressure and velocity in non-small cell lung cancer brain metastases treated with stereotactic radiosurgery**

Swinburne, et al.

# Appendix

## 1.1.1 CFM Mathematical Model

The fluid mechanics of a system are given by the Navier-Stokes hydrodynamic mass-balance equation (28).

$$\rho\left(\frac{\partial \mathbf{u}}{\partial t}+\ \mathbf{u}\cdot\nabla\mathbf{u}\right)=-\nabla p_i+\left[\nabla\cdot\mu\left(\nabla\mathbf{u}+(\nabla\mathbf{u})^{\mathrm{T}}-\frac{2\mu}{3}(\nabla\cdot\mathbf{u})\mathbf{I}\right)\right]+\frac{\mu}{K}\mathbf{u}+\mathbf{F}\quad [1]$$

The extracellular matrix is modeled as a porous medium of hydraulic conductivity $K_{\mathrm{H}}=K/\mu$. In equation [1], the interstitial fluid velocity (IFV) is described by the vector $\mathbf{u}$ (m/s), $p_i$ is interstitial fluid pressure (IFP) (Pa), $K$ is the permeability of the tissue ([$m^2$], describing the packing of the extracellular matrix), $\rho$ is interstitial fluid density ($kg/m^3$), and $\mu$ is interstitial fluid viscosity (Pa∗s). Additional volume forces acting on the fluid element may be represented as **F** (N); **I** and T are the notation for the identity matrix and the transpose of the operator.

Equation [1] simplifies in the case of an incompressible fluid ($\nabla\cdot\mathbf{u}=\mathbf{0}$), ignoring friction within fluid and exchange of momentum between fluid and solid phases. Fluid movement through extracellular extravascular (EES) is approximated with low-Reynolds Number flow (29) and modeled under assumption of steady-state velocity. The expression reduces to Darcy's Law where interstitial fluid velocity is given by the gradient in IFP ($\nabla p_i$) to describe bulk fluid movement:

$$\mathbf{u}=-K_{\mathrm{H}}\nabla p_i\qquad [2]$$

A dynamical continuity equation can then be composed according to the following system model: fluid enters EES via the vascular compartment ($\varphi_{\mathrm{v}}$, flux of blood delivery). In the human brain, there is no established lymphatic system of clearance, and we take the lymphatic flux term to be zero in both normal and tumor tissue:

$$\phi_{\mathrm{L}}=0,\ \textit{no lymphatic flux in normal tissue and tumor}\qquad [3]$$

Blood flow flux $\phi_{\mathrm{v}}$ across capillary walls is regulated according to Starling's Law:

$$\phi_{\mathrm{v}}=L_{\mathrm{P}}\frac{S}{V}\left(p_{\mathrm{V}}-p_{\mathrm{i}}-\sigma_{\mathrm{T}}(\pi_{\mathrm{V}}-\pi_{\mathrm{i}})\right)\quad [4]$$

Where $L_{\mathrm{P}}$ is the hydraulic conductivity of the capillary wall (or vessel permeability), *S/V* is microvascular surface area per unit volume, $p_{\mathrm{V}}$ is the blood pressure in the microvessel, $p_{\mathrm{i}}$ is interstitial fluid pressure; $\pi_{\mathrm{V}}$ is osmotic pressure in microvasculature, $\pi_{\mathrm{i}}$ is osmotic pressure in interstitial space, and $\sigma_{\mathrm{T}}$ is the osmotic reflection coefficient.

Combining equations [2] and [4], the continuity equation becomes:

$$\nabla\cdot\mathbf{u}=L_{\mathrm{P}}\frac{S}{V}\left(p_{\mathrm{V}}-p_{\mathrm{i}}-\sigma_{\mathrm{T}}(\pi_{\mathrm{V}}-\pi_{\mathrm{i}})\right)\qquad [5]$$

The final expression for the continuity equation [Eq. 5] in terms of the dependent variable interstitial pressure, $p_\mathrm{i}$:

$$-K_\mathrm{H}\nabla^2 p_i = \frac{K^\mathrm{trans}}{\langle K^\mathrm{trans}\rangle}\left[L_\mathrm{P}\frac{S}{V}\left(p_\mathrm{V} - p_\mathrm{i} - \sigma_\mathrm{T}(\pi_\mathrm{V} - \pi_\mathrm{i})\right)\right] - \frac{L_\mathrm{pL}S_\mathrm{L}}{V}(p_\mathrm{i} - p_\mathrm{L}) \qquad [6]$$

Where $\langle K^\mathrm{trans}\rangle$ represents mean $K^\mathrm{trans}$ values within the tumor; this term is used to account for heterogeneous fluid leakiness in the tumor (30). Estimates for physical parameters were selected in agreement with previous literature on modeling IFP in brain tumors (21,31–34).
